# Chiral twisting in a bacterial cytoskeletal polymer affects filament size and orientation

**DOI:** 10.1038/s41467-020-14752-9

**Published:** 2020-03-16

**Authors:** Handuo Shi, David A. Quint, Gregory M. Grason, Ajay Gopinathan, Kerwyn Casey Huang

**Affiliations:** 1 Department of Bioengineering, Stanford University, Stanford, CA 94305 USA; 2 Department of Physics, University of California at Merced, Merced, CA 95343 USA; 3 NSF-CREST: Center for Cellular and Biomolecular Machines, University of California at Merced, Merced, CA 95343 USA; 4 Department of Polymer Science and Engineering, University of Massachusetts, Amherst, MA 01003 USA; 5 Department of Microbiology and Immunology, Stanford University, Stanford, CA 94305 USA; 6 Chan Zuckerberg Biohub, San Francisco, CA 94158 USA

**Keywords:** Biopolymers in vivo, Cytoskeleton, Bacterial structural biology, Cellular microbiology

## Abstract

In many rod-shaped bacteria, the actin homolog MreB directs cell-wall insertion and maintains cell shape, but it remains unclear how structural changes to MreB affect its organization in vivo. Here, we perform molecular dynamics simulations for *Caulobacter crescentus* MreB to extract mechanical parameters for inputs into a coarse-grained biophysical polymer model that successfully predicts MreB filament properties in vivo. Our analyses indicate that MreB double protofilaments can exhibit left-handed twisting that is dependent on the bound nucleotide and membrane binding; the degree of twisting correlates with the length and orientation of MreB filaments observed in vitro and in vivo. Our molecular dynamics simulations also suggest that membrane binding of MreB double protofilaments induces a stable membrane curvature of similar magnitude to that observed in vivo. Thus, our multiscale modeling correlates cytoskeletal filament size with conformational changes inferred from molecular dynamics simulations, providing a paradigm for connecting protein filament structure and mechanics to cellular organization and function.

## Abstract (web summary)

The actin homolog MreB directs cell-wall insertion and maintains cell shape in many rod-shaped bacteria. Here, Shi et al. perform molecular dynamics simulations for MreB to extract mechanical parameters for inputs into a coarse-grained biophysical polymer model that predicts MreB filament properties.

## Introduction

The actin and tubulin families of cytoskeletal proteins constitute essential components of cellular physiology in virtually all bacteria, archaea, and eukaryotes. Despite structural similarities within each of the two families, their primary functions span a diverse range of processes, including cell morphogenesis^1^, division^2,3^, and DNA segregation^4^. In living bacteria, many of these cytoskeletal proteins form filaments that are highly dynamic. Structural tools such as X-ray crystallography and cryo-electron microscopy have elucidated various filament structures within the bacterial actin family, including antiparallel, straight double protofilaments of MreB^5^, single, polar polymers of FtsA^3^, and bipolar, antiparallel filaments of ParM^4^, suggesting that filament conformations are highly tunable and have been selected for particular physiological functions over evolutionary time. However, the links between the conformational dynamics of these proteins in vivo and the molecular mechanisms by which they organize within cells to regulate cell physiology remain unclear. Molecular dynamics (MD) simulations are a powerful tool for identifying protein structural dynamics and filament mechanics at atomic resolution, providing key information to map filament properties from the protein to the cellular scales.

One such cellular-scale property defined by a bacterial actin homolog is cell shape, which is ultimately dictated by the rigid cell wall, a highly cross-linked mesh of peptidoglycan. During growth, cells actively remodel their cell wall while robustly maintaining their shape^6^. In rod-shaped bacteria such as *Escherichia coli*, cell-wall synthesis during elongation is regulated by the widely conserved actin homolog MreB^7^, which dictates the pattern of insertion of new cell-wall material^8^ and thereby maintains rod shape^7,9^. Genetic depletion and chemical inhibition of MreB lead to misshapen cells and eventually cell lysis^10,11^. Many point mutations in MreB alter cell shape in subtle ways, such as changing cell width^12–14^, curvature^15^, or polar morphology^14,16^ without affecting viability. In *E. coli*, MreB forms short filaments that move along the cell periphery^1^, and the localization and movement of these filaments are correlated with cell width^17^. *E. coli* MreB movement is chiral, which is correlated with twisting of cell body during elongation^17^. Previous MD studies of *Thermotoga maritima* MreB (TmMreB) showed that ATP hydrolysis and polymerization affect MreB monomer conformation, which in turn regulates the bending of MreB dimers^18^. The bending of a TmMreB dimer was also altered in silico by binding to the membrane protein RodZ, which directly interacts with MreB and tunes cell shape^19^. In *E. coli*, MreB forms antiparallel double protofilaments^5^ that can deform membranes^20^, and the double protofilament conformation is essential for rod-shape maintenance in *E. coli*^5^. However, it remains obscure how molecular-level changes in MreB connect to the biophysics of the double protofilament structure, and to the spatial patterning and functions of MreB in vivo.

**Fig. 1:**
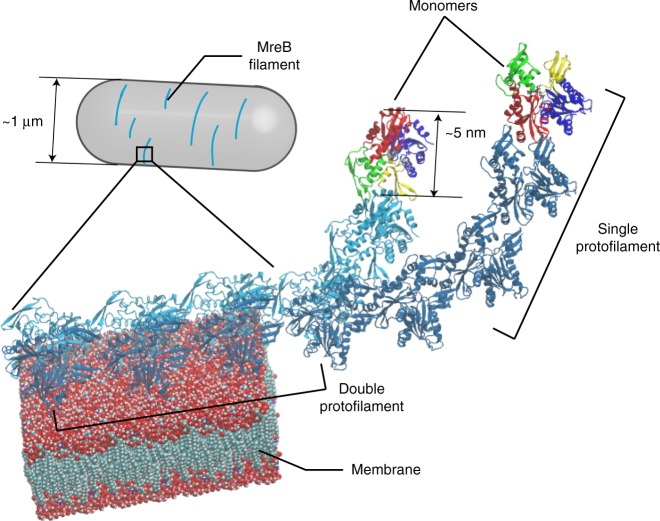
Assembly of MreB protofilaments. MreB monomers first polymerize into single protofilaments. Next, two antiparallel single protofilaments assemble into a double protofilament, with membrane-binding domains on the same side of the double protofilament^5^. Inside bacterial cells, short-MreB filaments bind the inner face of the plasma membrane, align approximately circumferentially, and rotate around the long cell axis to guide cell-wall insertion and to determine rod-like shape and size.

In this study, we exploit the recent solution of a crystal structure of a double protofilament of *Caulobacter crescentus* MreB^5^ (CcMreB) to explore the connection between MreB structural dynamics in silico and filament conformation in vivo. We perform all-atom MD simulations for each step during CcMreB filament assembly (Fig. 1), from monomers to single protofilaments, and then to double protofilaments without and with a membrane. Simulations of double protofilaments suggest a left-handed twisting conformation in ATP-bound double protofilaments. In our simulations, the degree of twisting was reduced when the double protofilaments were bound to ADP or a membrane, and binding to a membrane induced membrane curvature mimicking that of bacterial cells. We use our MD simulations to extract parameters relevant for coarse-grained analyses of finite, membrane-bound MreB double protofilaments, and explore a connection between intrinsic twist and filament length. Our model indicates that larger intrinsic twist would increase the buildup of mechanical strain in membrane-bound filaments and thereby decrease the maximum filament length, a prediction consistent with our in vivo observations with *E. coli* MreB mutants. Taken together, our results reveal a correlation between inferred molecular-scale features of MreB and experimental observations in *E. coli*. We propose that underlying this correlation is the ability of protofilament twist state, and its deformations at the intrafilament scale, to sense local curvatures along the cell membrane on length scales ~100-fold larger than protein monomers.

## Results

### MreB monomer opening is nucleotide and polymerization dependent

**Fig. 2:**
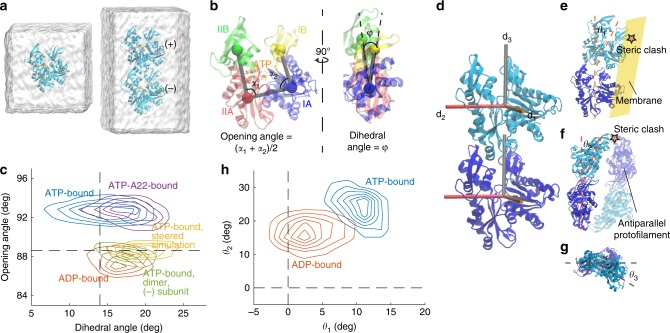
MreB monomer and dimer conformations are nucleotide dependent. **a** Simulated systems of an MreB monomer (left) and a single protofilament with two subunits (“2 × 1 protofilament”, right). Each MreB subunit is bound to a nucleotide, with the whole system surrounded by a water box. In the 2 × 1 single protofilament, we refer to the top and bottom MreB subunits as the (+) and (−) subunits, respectively. **b** Definitions of opening angle and dihedral angle for an MreB monomer, with the centers-of-mass of the four subdomains shown as colored spheres. **c** Contour density plot of the distributions of opening and dihedral angles for each simulation system from the last 40 ns of the simulation. MreB subunits essentially adopted one of two conformations in simulations. ATP-bound MreB monomers exhibited large opening angles in the presence (purple) and absence (blue) of A22, while an ADP-bound monomer (red) and the (−) subunit of an ATP-bound dimer (green) had smaller opening angles. Steering of the opening angle of an ATP-bound monomer to its value in the crystal structure (yellow) mimicked the conformation of an ADP-bound monomer. Dashed lines denote the values of the opening and dihedral angles in the crystal structure (PDB ID: 4CZF). **d** An MreB dimer from a single protofilament, with three axes (**d**_**1**_, **d**_**2**_ and **d**_**3**_) overlaid on each subunit that were used to compute the degree of bending and twisting between them. **e**) Illustration of *θ*_1_, with positive *θ*_1_ denoting bending toward the membrane surface (yellow). Non-zero *θ*_1_ leads to a steric clash with the membrane surface. **f** Illustration of *θ*_2_, with positive *θ*_2_ denoting bending toward the inter-protofilament interface. The paired antiparallel protofilament is shown in semi-transparency. Non-zero *θ*_2_ leads to a steric clash with the paired protofilament. **g** Illustration of *θ*_3_ from the top of a protofilament, with positive *θ*_3_ denoting left-handed twisting. **h** Contour density plot for the distributions of *θ*_1_ and *θ*_2_ from the last 40 ns (200 frames) of the simulations, with both ATP- and ADP-bound single protofilaments bending toward the membrane side and toward the inter-protofilament interface. An ATP-bound single protofilament exhibited more substantial bending in both directions than an ADP-bound single protofilament. Dashed lines denote the respective angles in the crystal structure.

To study the first step of MreB oligomeric assembly (Fig. 1), we performed all-atom MD simulations of MreB as a monomer and as a dimer in a single protofilament (Fig. 2a, Methods). All simulations were initialized from the crystal structure of the CcMreB single protofilament (PDB ID: 4CZF)^5^. The amphipathic helix (residues 1–8) missing from the crystal structure was added back using the Molefacture Protein Builder plugin in VMD (Methods), and water and neutralizing ions (Na^+^ or Cl^−^) were added to the simulation system. By analogy with actin, we refer to the two subunits in an MreB dimer as the (+) and (−) ends (Fig. 2a, right). The four subdomains were defined by aligning the MreB structure to that of actin, with the nucleotide bound in the center of the four subdomains (Fig. 2b)^5^.

In our simulations of an ATP-bound MreB monomer, we observed a rapid opening of subdomains IB and IIB, exposing the ATP-binding pocket. We quantified conformational changes by measuring the angles formed by the centers-of-mass of the four subdomains, defining an in-plane opening angle and an out-of-plane dihedral angle (Fig. 2b). The ATP-bound MreB monomer adopted a more open state, with an opening angle of ~92° at the end of an 80-ns simulation, compared to the ~88° opening angle of an ADP-bound MreB monomer (Fig. 2c, Supplementary Fig. 1a). The dihedral angle was slightly smaller in an ATP-bound monomer than in an ADP-bound monomer (Fig. 2c, Supplementary Fig. 1b), consistent with the larger dihedral angle in the crystal structure of CcMreB bound to ADP (PDB ID: 4CZL) versus CcMreB bound to AMP-PNP (PDB ID: 4CZM) (Supplementary Fig. 1c). This result qualitatively differed from our previously reported MD simulations using TmMreB, in which ATP-bound TmMreB exhibited a larger dihedral angle than ADP-bound TmMreB but a similar opening angle^18^. To interrogate this difference, we performed new simulations using ATP-bound TmMreB, and obtained results consistent with our previous study (Supplementary Fig. 1d, e)^18^. Therefore, although CcMreB and TmMreB are structurally similar, they likely adopt different conformations upon nucleotide binding. Such observations suggest that CcMreB and TmMreB may undergo different conformational changes during polymerization, which potentially relates to the polymeric differences observed in vitro wherein TmMreB formed straighter protofilaments on rigid lipid tubes than CcMreB^5^.

By contrast to the open conformation of an ATP-bound monomer, the (−) subunit in an ATP-bound MreB dimer maintained a closed conformation resembling the ADP-bound monomer (Fig. 2c), a conformation similar to subunits within a CcMreB protofilament crystal structure (Supplementary Fig. 1c)^5^. We calculated opening and dihedral angles for all published CcMreB crystal structures^5^, and found that monomeric structures have larger opening angles than polymerized structures (Supplementary Fig. 1c), supporting our conclusion that polymerization closes MreB.

Motivated by previous findings relating MreB conformation to ATP-binding pocket stability^18^, we quantified ATP-binding pocket stability by calculating the buried solvent-accessible surface area (SASA) between MreB and ATP (Methods). Buried SASA quantifies the surface area of an ATP-MreB interface (Supplementary Fig. 1f), and thus a larger buried SASA indicates a more stable ATP-binding pocket. The buried SASA in an ATP-bound CcMreB monomer decreased coincident with increases in opening angle (Supplementary Fig. 1g, h), and ATP-bound TmMreB exhibited similar decreases (Supplementary Fig. 1g). By contrast, the (−) subunit of an ATP-bound CcMreB dimer maintained high buried SASA, indicating that its ATP-binding pocket remained stable. To verify that the buried SASA of ATP is related to the opening angle, we performed steered simulations of an ATP-bound CcMreB monomer in which we constrained the opening angle to the crystal structure value of ~89°. Although the dihedral angle opened slightly in the steered simulation (Supplementary Fig. 1a, b), the buried SASA of ATP remained high (Supplementary Fig. 1g). Similarly, in our TmMreB monomer simulations, we observed a similar reduction in buried SASA when TmMreB opened (Supplementary Fig. 1i). Taken together, our simulations suggest that CcMreB monomers adopt distinct open and closed conformations; ATP-bound CcMreB monomers prefer the open state but close upon polymerization. The closed state may facilitate ATP hydrolysis by increasing the stability of the ATP-binding pocket.

We next asked how MreB conformation is affected in silico by the MreB inhibitor S-(3, 4-dichlorobenzyl) isothiourea (A22) by performing MD simulations with MreB bound simultaneously to both ATP and A22 (Methods). Although A22 is known to perturb cell morphology in vivo by targeting the active site of MreB^15,21^, the molecular mechanism of action is still obscure. In our simulations, A22 did not affect the MreB monomer opening angle, and only slightly increased the dihedral angle (Fig. 2c). We further quantified the stability of the ATP-binding pocket using buried SASA, and found that A22-ATP-bound MreB exhibited similar dynamics in buried SASA as ATP-bound MreB (Supplementary Fig. 1g). Thus, our results suggest that A22 does not directly affect MreB monomer conformation and is unlikely to alter the ATP-binding pocket, consistent with other studies proposing that A22 blocks phosphate release rather than inhibiting ATP hydrolysis^5,22^.

### Bending of a CcMreB single protofilament is nucleotide dependent

We next sought to study the conformational changes in single protofilaments with two CcMreB subunits (“2 × 1 protofilaments”) by analyzing the relative movements of the (+) and (−) subunits in the dimer (Fig. 2a–g). We simulated CcMreB 2 × 1 protofilaments with both subunits bound to ATP or ADP, and quantified their relative orientation changes by calculating the Euler angles that characterize the three orthogonal modes of rotation around the **d**_**1**_–**d**_**3**_ axes (Fig. 2d). The membrane-binding interface is on the right side of the protein (Fig. 2e). Thus, *θ*_1_ and *θ*_2_ characterize bending into the membrane surface and inter-protofilament surface, respectively (Fig. 2e,f), and *θ*_3_ characterizes twisting along the protofilament (Fig. 2g). We defined all three Euler angles to be zero in the crystal structure (Fig. 2d). Binding a double-protofilament to the inner surface of a cylinder favors a compatible geometry: a negative *θ*_1_ and a nearly zero *θ*_2_ to avoid steric clashes (Fig. 2e, f). We found that the largest changes in our simulations of single protofilaments occurred in the bending angles (Fig. 2h, Supplementary Fig. 1j, k), whereas no systematic protofilament twisting was observed (Supplementary Fig. 1l). The bending angles were also nucleotide-dependent, with ATP-bound protofilaments exhibiting larger bending angles than ADP-bound protofilaments (Fig. 2h), consistent with our previously reported results in TmMreB^18^.

Considering the double protofilament structure and the membrane-binding interface (Fig. 2e, f), both bending angles observed in our CcMreB 2 × 1 protofilament simulations are unlikely to occur in a double-protofilament architecture. A nonzero *θ*_2_ would require differential compression/stretching of the protofilament (Fig. 2f) and likely cause shear failure of inter-protofilament interactions. Positive *θ*_1_ corresponds to bending toward the membrane surface (Fig. 2e), whereas in vitro experiments indicate that MreB filaments bend away from the membrane^5^. Therefore, although single-protofilament simulations demonstrate the molecule’s capacity for nucleotide-dependent conformations, simulations of double protofilament conformations and consideration of membrane binding are critical for revealing MreB structural dynamics that are relevant in vivo.

### MreB double protofilament twisting is membrane dependent

**Fig. 3:**
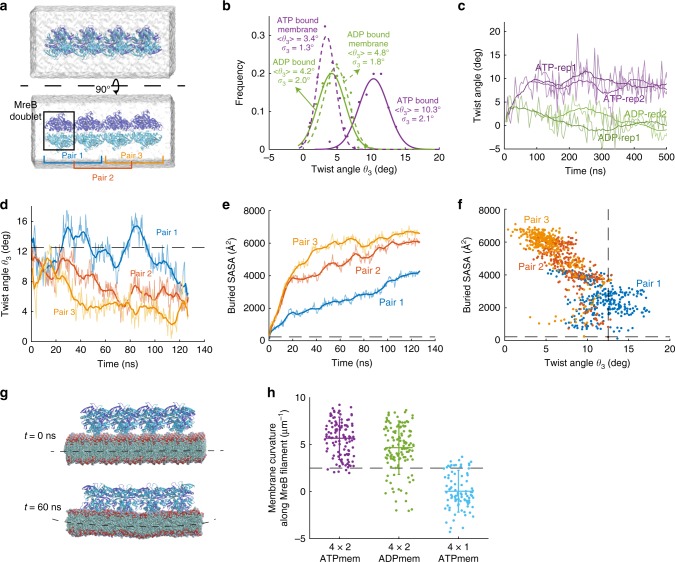
Membrane-binding decreases MreB filament twist and induces membrane curvature. **a** Simulated system of a 4 × 2 MreB double protofilament in water. The system consists of four MreB doublets (eight subunits), surrounded by a water box. **b** Distribution of twist angles in simulated systems at equilibrium. The ATP-bound 4 × 2 protofilament displayed a large twist angle, which decreased when the 4 × 2 protofilament bound the membrane. Membrane binding did not substantially affect the twist angle of ADP-bound protofilaments. Solid dots are histograms from the last 40 ns (200 frames) of each simulation, and curves are Gaussian fits of the histograms. **c** The twist angles for ATP- and ADP-MreB in (**b**) remained stable and easily distinguished between the two nucleotide-binding states in simulations longer than 500 ns. **d** Twist angles decreased over time when a pre-twisted 4 × 2 protofilament was placed close to a membrane patch. Untwisting occurred first in Pair 3, then propagated to Pair 2 and Pair 1. The dashed line shows the initial twist angle in Pair 2. **e** Buried SASA of the membrane-binding interface for the twisted protofilament. Higher buried SASA indicates stronger membrane interaction. Similar to the changes in twist angles, the buried SASA increased first in Pair 3, then in Pair 2 and Pair 1. The dashed line is the initial buried SASA for Pair 2. **f** Scatter plot of buried SASAs and twist angles in the simulation analyzed in (**d**, **e**). Each dot represents the values for a certain pair at a particular time point, and dashed lines are the initial values for Pair 2. Buried SASA and twist angle were highly correlated (Pearson’s *r* = 0.79, *p* < 10^−10^, Student’s *t* test). **g** In a typical membrane simulation with 4 × 2 protofilaments, the membrane started flat (top) and ended up curved toward MreB (bottom). **h** Values of induced membrane curvature in 4 × 2 and 4 × 1 protofilament simulations. Only the 4 × 2 protofilaments induced non-zero membrane curvatures. The dashed line represents the in vivo reference value for a rod-shaped cell with width 0.8 µm. Data points represent the mean ± S.D. for the last 40 ns of each simulation.

We next performed MD simulations of MreB double protofilaments, each containing four MreB doublets (a 4 × 2 protofilament, Fig. 3a). Simulations were performed with all MreB subunits bound to ATP or ADP (Fig. 3a, Methods), and at least two replicate simulations were performed for all systems. Equilibrium was reached after ~100 ns of simulation. The interactions within MreB doublets remained stable, while the doublets moved with regard to their neighbors. Therefore, we quantified the relative rotational movements for neighboring doublet pairs in the double protofilaments, using the same coordinate system as the 2 × 1 protofilaments (Fig. 2d–g). To minimize boundary effects, we first focused on the middle doublet (Pair 2; Fig. 3a). As expected, bending of each protofilament was dramatically different in a double protofilament versus a single protofilament. *θ*_1_ values were smaller in magnitude and were generally negative (Supplementary Fig. 2a), indicating slight bending away from the membrane, and *θ*_2_ decreased to approximately zero (Supplementary Fig. 2b). Instead of bending along *θ*_2_, which would disrupt the symmetry and stability of a double protofilament, twisting (*θ*_3_) was prominent in the double protofilament (Fig. 3b, Supplementary Fig. 2c). In all 4 × 2 protofilament simulations, left-handed twisting was observed. Interestingly, in water, an ATP-bound double protofilament twisted more (10.3 ± 2.1°, mean ± S.D. from Gaussian fitting of last 40 ns of simulation) than an ADP-bound double protofilament (4.2 ± 2.0°).

To determine the stability of these conformations, we performed longer simulations extending to ~500 ns. The twist angles in ATP- and ADP-bound double protofilaments remained distinct and relatively stable over this long time scale (Fig. 3c). To confirm that our observations on bending and twisting were not artefacts due to limited filament size, we performed a larger simulation with eight ATP-bound MreB doublets in water (an 8 × 2 protofilament). In this 60-ns simulation, changes in bending and twist angles matched our observations in 4 × 2 protofilaments (Supplementary Fig. 2d–f, Supplementary Movie 1). To verify that the double-protofilament twist was not unique to MreB from *C. crescentus*, we constructed a homology model of *E. coli* MreB (Methods), and found that EcMreB exhibited quantitatively similar left-handed twisting in simulation (Supplementary Fig. 2g). Thus, higher-order oligomerization can dramatically alter the biophysical properties of MreB filaments.

The twisted double protofilament conformation is not compatible with experimental observations of straight MreB filaments binding to a rigid, flat membrane surface^5^. To address this incompatibility, we performed MD simulations of 4 × 2 protofilaments in the presence of a membrane patch. The membrane patch in our simulations was composed of all-atom phosphatidylethanolamine molecules neutralized by NaCl. Membrane binding reduced the twist of ATP-bound double protofilaments but did not affect the less-twisted ADP-bound structures (Fig. 3b). To test the hypothesis that membrane binding suppresses twisting in ATP-bound double protofilaments, we took the twisted protofilament structure from the end of an ATP-bound 4 × 2 protofilament simulation in water, and placed it ~10 Å away from a membrane patch. Within 120 ns, the filament gradually untwisted from one end to the other (Fig. 3d, Supplementary Movie 2), effectively “zippering” into the membrane. The decrease in twist angle from each doublet was accompanied by an increase in buried SASA at the protein-membrane interface, indicative of stronger MreB–membrane interactions (Fig. 3e). Pair 1 did not reach as high buried SASA values as the other pairs over the course of this simulation; nonetheless, the dynamics between twist angle and buried SASA correlated well (Fig. 3f), suggesting that membrane binding directly suppresses twisting in ATP-bound MreB double protofilaments. The results from these membrane simulations are consistent with in vitro observations in which MreB filaments bound to membrane surfaces without substantial twist^5,20,23^.

We further asked whether membrane binding alters the stability of the double protofilament conformation, as quantified by the distances between the interacting V118 residues within each MreB doublet (Supplementary Fig. 2h), which are essential for forming a double-protofilament structure^5^. For both ATP- and ADP-bound double protofilaments, our simulations in water exhibited increased distances between V118 residues in the first 60 ns (Supplementary Fig. 2i), suggesting a destabilized double protofilament interface. In contrast, membrane-associated simulations maintained short V118 distances (Supplementary Fig. 2i), indicating more stable double protofilaments. Therefore, membrane binding potentially stabilizes the double-protofilament structure.

### Double protofilaments induce membrane curvature

The distinct structures of MreB double protofilaments when bound or unbound to a membrane patch and the lack of complete untwisting when membrane-bound (Fig. 3b) indicated that membrane binding introduced strain into the MreB filaments that may deform the membrane itself. In our simulations, the membrane started flat, but after 60 ns, the membrane bent toward the MreB protofilaments (Fig. 3g). To validate that the observed membrane curvature changes were related to the conformation of double protofilaments, we performed simulations of a 4 × 1 protofilament bound to ATP in the presence of a membrane patch as a control. The membrane patch bound to the 4 × 1 protofilament did not exhibit a characteristic curvature throughout the simulation (Fig. 3h). Thus, only double MreB protofilaments induce stable curvature in the membrane, consistent with a previous finding that MreB needs to form double protofilaments to function in vivo^5^.

### Mutations in MreB and binding of RodZ modulate intrinsic twist

**Fig. 4:**
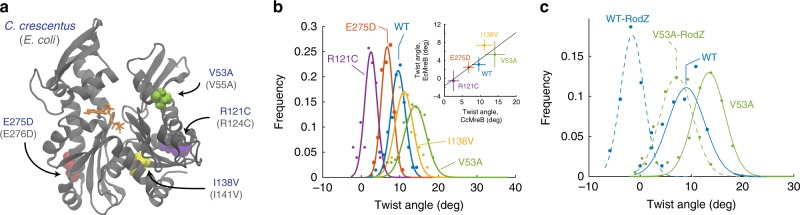
MreB mutants alter twist angles in simulation. **a** Mutations in MreB investigated via MD simulations mapped onto the CcMreB crystal structure. These mutations were previously identified to alter cell shape^14,19^, and are conserved between CcMreB (residue numbers in blue) and EcMreB (residue numbers in light gray). Colored spheres: mutated residues. Orange: ATP molecule. Gray: MreB protein structure. **b** Distributions of twist angles in simulations of CcMreB mutants. All systems started with zero twisting. E275D and R121C twisted less than wild-type MreB, while V53A and I138V twisted more. Dots are histograms from the last 40 ns of each simulation, and curves are Gaussian fits of the histograms. Inset: MD simulations for mutants in an EcMreB homology model also exhibited left-handed twisting, and the twist angles were correlated with those of the corresponding CcMreB mutants. Data points are mean ± S.D. from Gaussian fits. **c** Distributions of twist angles with and without RodZ binding. For wildtype and the V53A mutant, binding of the cytoplasmic tail of RodZ decreased twisting. The effect of RodZ binding was approximately additive to the effects of MreB mutation, such that the V53A-RodZ system twisted more than the WT-RodZ system. Dots are histograms from the last 40 ns of each simulation, and curves are Gaussian fits of the histograms.

We hypothesized that since many MreB mutations alter cell shape, they potentially also alter the intrinsic twist and membrane interactions of double protofilaments. We identified four MreB mutants that were reported to cause a range of alterations to *E. coli* cell shape, with the corresponding residues conserved between CcMreB and EcMreB: R124C^24^, E276D^19^, A55V^14^, and I141V^14^. The four mutated residues are spread across the MreB structure (Fig. 4a), and thus potentially alter MreB function in different manners.

We first performed all-atom MD simulations for each of the corresponding CcMreB mutants bound to ATP in a 4 × 2 protofilament configuration in water. All mutants exhibited similar bending (Supplementary Fig. 3a, b), but differed widely in twist angles compared to wild-type CcMreB: E275D (E276D in EcMreB) and R121C (R124C in EcMreB) twisted less than wildtype, whereas V53A (V55A in EcMreB) and I138V (I141V in EcMreB) exhibited more twist (Fig. 4b, Supplementary Fig. 3c). To determine whether these mutations are also likely to alter twist angles in *E. coli*, we performed 4 × 2 protofilament simulations using an EcMreB homology model (Methods), and found that the twist angles in EcMreB mutants were highly correlated with those of the corresponding CcMreB mutants (Pearson’s *r* = 0.85, *p* = 0.05, two-tailed Student’s *t* test), despite overall lower twist angles in EcMreB (Fig. 4b, inset). Therefore, we predict that the mutations alter the intrinsic twist similarly in CcMreB and EcMreB. For later simulations, we used CcMreB structures as they exhibited a larger range of twist angles across mutants and hence provided better resolution in MD simulations.

We next asked whether these mutants also exhibit differential twisting when membrane-bound by simulating 4 × 2 protofilaments of R121C and V53A in proximity to a membrane patch. These two mutants were selected because they exhibited the smallest and the largest intrinsic twisting in our MD simulations in water, respectively (Fig. 4b). Despite the large differences in intrinsic twisting of these mutants in water, they behaved similarly when bound to a membrane, wherein twist angles were suppressed down to similar levels as wild-type MreB (Supplementary Fig. 3d–f). Therefore, genetic perturbations can modulate the intrinsic twist of MreB double protofilaments without disrupting the ability of MreB to form stable membrane-binding complexes or to maintain rod-shaped growth. However, to untwist a highly twisted filament costs more energy compared to a less-twisted filament, which potentially alters the higher-order conformation and/or orientation of membrane-bound MreB in vivo, as we will address below.

To determine whether the mutations affect the ability of MreB to interact with the membrane, we performed simulations of membrane-bound MreB monomers. All mutants exhibited similar behavior, particularly the buried SASA at the membrane-binding interface (Supplementary Fig. 3j). From the buried SASA dynamics, we estimated that the membrane binding potential for an MreB monomer is ~10 *k*_B_*T* (Methods).

The membrane protein RodZ directly interacts with MreB^25^ and is essential for rod-shape maintenance^26^. *E. coli* cells actively tune the stoichiometry of MreB and RodZ as a function of growth rate and growth phase^19,27^, and changes in the MreB:RodZ ratio alter the localization pattern of MreB and cellular dimensions^19^. We previously showed that RodZ binding and MreB mutations that complement the loss of rod-like shape in ∆*rodZ* cells both alter the mechanics of single TmMreB protofilaments in vivo^19^. Therefore, we hypothesized that RodZ binding also affects MreB double-protofilament conformations. We constructed a homology model for the cytoplasmic tail of *C. crescentus* RodZ from the cocrystal structure of *T. maritima* RodZ and MreB (PDB ID: 2UWS)^25^, and aligned it to the RodZ-binding interface for each of the subunits in a 4 × 2 CcMreB protofilament (Methods). The ratio of MreB and RodZ levels in *E. coli* cells varies from ~10:1 to ~4:1 depending on growth conditions^19^, and hence MreB filaments are likely to experience a range of RodZ stoichiometries; modeling an MreB protofilament with partially-bound RodZ can cause substantial boundary effects. Therefore, we simulated MreB protofilaments in water with each MreB monomer bound to RodZ, and postulated that the in vivo effects of RodZ should be an intermediate between no RodZ and full RodZ binding. All-atom MD simulations showed that while RodZ binding did not substantially change either of the bending angles in a double protofilament (Supplementary Fig. 3g, h), it significantly reduced the twist angle of MreB (Fig. 4c, Supplementary Fig. 3i). Thus, our simulations suggest that RodZ abundance actively regulates MreB filament conformation in vivo^19^.

Since MreB mutations and RodZ binding both alter the twisting of an MreB double protofilament, we performed MD simulations for an MreB mutant (V53A) bound to the cytoplasmic tail of RodZ; the V53A 4 × 2 protofilament in the absence of RodZ exhibited the largest twisting in our simulations (Fig. 4b). Simulations of RodZ bound to a V53A 4 × 2 protofilament exhibited partially suppressed twisting (Fig. 4c, Supplementary Fig. 3i), with an average twist slightly lower than that of wild-type MreB in the absence of RodZ (Fig. 4c). The additivity of effects on twisting suggests that RodZ and MreB mutations can alter double protofilament twist in orthogonal manners. Therefore, although regulatory proteins are likely to modulate the intrinsic twisting in MreB double protofilaments, they likely shift the absolute twist but keep the order of twist angles across mutants.

### MreB twist in silico predicts filament conformation in vivo

**Fig. 5:**
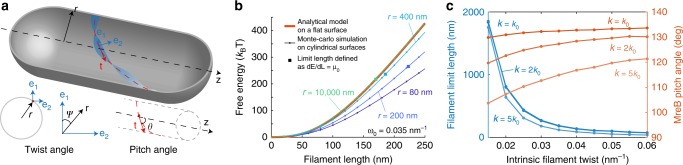
Coarse-grained model connects filament twist with conformational changes in vivo. **a** Schematic of our coarse-grained model. The MreB filament (blue) is bound to the inside of a cylindrical cell body (gray) with radius (*r*). The centerline of the filament is drawn in red, with **t** being the tangent vector. **e**_**1**_ and **e**_**2**_ are the material frame coordinates along the filament. Local twist angle *ψ* is defined as the angle between **e**_**1**_ and the unit vector **r**. Local pitch angle *θ* is defined as the angle between tangent vector **t** and the cylindrical centerline. **b** Analytical results and Monte Carlo simulations for finite MreB filaments predict that smaller membrane radius (*r*) reduces the free energy and increases the filament limit length (solid squares). When the membrane radius is reduced to 80 nm, the model predicts that the filament can extend without an energetic limit. Each data point represents the mean over 20 independent Monte Carlo simulations. The variation across repeats are smaller than the size of the data symbols. **c** The coarse-grained model predicts that filaments with larger intrinsic twisting have shorter limit length. Similarly, the orientation of the filament is predicted to deviate more from 90° as the intrinsic twist increases. Increasing the intrinsic curvature *k* did not affect the limit length, but reduced the pitch angle to be closer to 90°. Data points represent mean ± S.D. from 20 independent Monte Carlo simulations. The error bars are smaller than the size of the data symbols.

How does the intrinsic twist of MreB double protofilaments affect their conformation in vivo? To answer this question, we developed a coarse-grained model in which an MreB double protofilament is represented as a beam, with its bending and twist stiffness extracted from our all-atom MD simulations (Methods). Considering that the large turgor pressure across the bacterial cell envelope (~1 atm^28^) forces the membrane to adopt a shape matching that of the cell wall, we treated the membrane as a rigid cylindrical surface. We first considered the case of binding to a flat membrane surface, and minimized the free energy of an MreB beam of different lengths *L* with intrinsic twist and curvature (Methods, Fig. 5a). Intuitively, in the presence of an interaction between the filament and the membrane, a twisted filament can gain binding energy by untwisting so that more of its membrane-binding interface can contact the membrane, but the untwisting process also accumulates bending and twisting energy. Therefore, free energy increases non-linearly with filament length *L* (Fig. 5b, orange curve). When the energetic cost of further extension of the filament equals the polymerization energy, the filament reaches its limit length. For MreB, we estimate the free energy to polymerize a monomer pair to be 10 *k*_B_*T* based on measurements from actin (Methods)^29^, which corresponds to a polymerization energy per unit length of *µ*_0_ = 2 *k*_B_*T* nm^−1^. In the case of a flat membrane surface, this value of *µ*_0_ corresponds to a limit length of ~160 nm (Fig. 5b), which is in quantitative agreement with in vitro observations of CcMreB filaments bound to rigid membrane surfaces^5^.

We next used Monte Carlo simulations to calculate the dependency of free energy on filament length on a curved membrane with radius *r* (Methods). When *r* = 10,000 nm, much larger than the length scale of an MreB filament, our Monte Carlo calculations agreed quantitatively with the analytical solution (Fig. 5b, green curve). For smaller *r*, the cylindrical surface allows the filament to retain more twist while remaining membrane associated^30^, and hence the free energy *E* at a given length *L* decreases, which increases the limit length (Fig. 5b, solid squares). Interestingly, when *r* = 80 nm or smaller, d*E*/d*L* was always <*µ*_0_ (Fig. 5b, Supplementary Fig. 4a, b), suggesting that for sufficiently small membrane radius MreB filaments can extend without energetic limitations. This prediction is consistent with in vitro observations of long MreB filaments in tubulated liposomes with radius ~80 nm^23^.

Our coarse-grained model predicts that the limit length of MreB filaments should decrease with increasing intrinsic twist (Fig. 5c). Similarly, the local pitch angle *θ* (Fig. 5a) balances between filament bending and twisting: with a pitch angle of 90°, the filament fully untwists but largely preserves bending; when the pitch angle deviates from 90°, the filament reduces bending while remaining somewhat twisted. Therefore, from an energetic point of view, our coarse-grained model predicts that the intrinsic twisting in an MreB filament causes its orientation to deviate from the perfect circumferential direction (pitch angle *θ* = 90°) (Fig. 5c). We further performed sensitivity analyses by altering the parameters that affect filament conformation^30^. For instance, by varying the intrinsic curvature *k*, we found that the limit-length predictions were largely unaffected, whereas larger values of *k* led to pitch angles closer to 90° (Fig. 5c). Similarly, altering the bending modulus (*C*) changed the pitch angle but not limit length (Supplementary Fig. 4c), while increasing the twist modulus (*K*) or the membrane binding potential (*V*) altered the limit length without substantially affecting the pitch angle (Supplementary Fig. 4d, e). Notably, despite variation in the predicted values across parameters, our model generally predicts that larger intrinsic twist leads to shorter filaments with larger pitch angles. Therefore, although many cellular factors such as MreB regulators could affect the intrinsic twist and/or mechanical properties of MreB, the trend of filament length and orientation predicted by our model (Fig. 5c) should still hold in vivo.

**Fig. 6:**
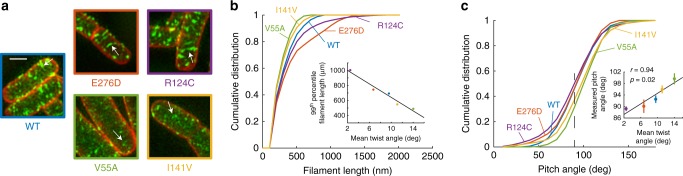
MreB twist angles predict filament length and orientation in vivo. **a** Structured illumination microscopy of wildtype and the four EcMreB mutants in Fig. 4a constructed in *E. coli* cells with a sandwich fusion of msfGFP to MreB. Images are maximum projections of a *z*-stack, with red (membrane dye FM 4-64FX) and green (MreB-msfGFP) channels merged. White arrows highlight typical MreB filaments in each strain. Scale bar is 1 µm. **b** The cumulative distributions of MreB-msfGFP fluorescence filament length across mutants trend with their predicted intrinsic twist angles in Fig. 4b. The V55A and I141V strains had shorter filaments than wildtype, and the E276D and R124C strains contained longer filaments. MreB filaments were defined as continuous regions with high msfGFP signal on the cylindrical cell body with area larger than the diffraction limit. *n* > 1000 filaments were measured for each strain. Inset: the 99th percentile of filament length in each strain was highly correlated with the mean twist angle from Fig. 4b (Pearson’s *r* = −0.98, *p* = 0.004, Student’s *t* test, *n* = 5 strains), providing experimental validation of the coarse-grained model. **c** Cumulative distributions of MreB filament pitch angle. The V55A and I141V strains had larger pitch angles than wildtype, while E276D and R124C had smaller pitch angles that were closer to 90°. The pitch angle was defined as the angle between the main axis of each fluorescent patch and the long axis of the cell. Inset: the experimentally measured pitch angle was highly correlated with the mean twist angle from Fig. 4b (Pearson’s *r* = 0.94, *p* = 0.02, Student’s *t* test, *n* = 5 strains). Data points are mean ± S.E.M. for *n* > 1000 patches in each strain.

To test the predictions of our coarse-grained model, we experimentally constructed *E. coli* strains expressing the MreB mutants (Fig. 4a) with a sandwich fusion of monomeric super-folder green fluorescent protein (msfGFP)^31^ as the sole copy of MreB. To quantify the shape and size of the MreB filaments, we imaged each strain using super-resolution structured illumination microscopy (Methods). In wild-type cells, MreB formed short filaments with lengths around ~200–300 nm (Fig. 6a). The E276D and R124C mutants clearly contained much longer filaments that spanned roughly half the cell periphery, whereas V55A and I141V had very short MreB filaments (Fig. 6a). We quantified the size of each MreB patch by reconstructing the 3D surface of each cell and extracting high-GFP regions from the flattened surface. Each connected region with high-GFP intensity was considered one MreB filament, and its length was estimated based on the major axis of this region (Methods). Indeed, E276D and R124C had longer MreB filaments than wildtype, and V55A and I141V had shorter filaments (Fig. 6b). We used the 99^th^ percentile of MreB filament length as an approximation for filament limit length. Although many in vivo factors affect MreB twist angle, nevertheless intrinsic twist angles for the mutants in our all-atom MD simulations (Fig. 4b) were highly negatively correlated with the experimentally measured filament limit lengths (Fig. 6b, inset), consistent with the predictions of our coarse-grained model (Fig. 5c). Similarly, we calculated the pitch angle of each MreB patch from the microscopy images (Fig. 6c) and observed that MreB filament orientation positively correlated with intrinsic twist (Fig. 6c, inset): a larger intrinsic twist led to a larger deviation from circumferential orientation. Taken together, our microscopy results correlated strongly with the predictions of our coarse-grained model that the intrinsic twist of MreB double protofilaments affects filament limit length and orientation in vivo.

## Discussion

Here, we used MD simulations to propose a twisted double-protofilament conformation of CcMreB (Fig. 3b, c) and EcMreB (Supplementary Fig. 2g). Our results indicate that twisting would be regulated by various factors, including the binding nucleotide (Fig. 3b, c), the membrane (Fig. 3c), genetic perturbations (Fig. 4b), and regulatory proteins (Fig. 4c). While previous MD simulations of TmMreB provided insights into the structural properties of MreB at the monomer and single-protofilament levels^18,19^, our analyses predict that the twist only occurs in the context of a double protofilament. Using a coarse-grained model, we further linked the intrinsic twisting of MreB filaments to their limit length and orientation when bound to the membrane (Fig. 5b, c). Since EcMreB shares a higher sequence similarity with CcMreB (62%) than with TmMreB (52%), our MD studies in CcMreB also permit more versatile mutagenesis studies linking simulations to experimental measurements in *E. coli*, from which we tested the predictions of our coarse-grained model in vivo with fluorescence measurements of MreB mutants predicted to have altered twist (Fig. 6).

Twisting of MreB breaks symmetry and introduces chirality. Chirality is a common feature of biological systems. During bacterial growth, chirality has been observed at the population^32^ and single-cell^17,33^ levels, and can be altered by perturbing MreB or other components of the cell-wall synthesis machinery^17^. Previous in silico studies of single MreB protofilaments calculated twist angles very close to zero, similar to our single-protofilament simulations (Supplementary Fig. 1l). By contrast, our double-protofilament simulations indicate the potential for a large and stable left-handed twist, highlighting a potential tunable source of chirality at the molecular level (Fig. 3c, Fig. 4b) that may be related to single-cell twisting during *E. coli* growth^17,33^. Further understanding of the emergence of asymmetry and MreB twisting will benefit from recent advances in protein design^34^. The design of MreB mutants with various intrinsic twists can be directly tested in vivo to further probe the connections between molecular twisting and single-cell physiology. More broadly, this study suggests a potential generic mechanism whereby shape incompatibility of a filament and surface provides a means to “measure” size and geometry of substrates using nm-scale protein building blocks. The general rules underlying filament twisting can be utilized to construct synthetic architectures in cells that have variable binding interfaces, mechanical properties, and, as suggested by our results with MreB, tunable lengths and orientations when bound to a membrane. We note the possibility that membrane binding could induce a major conformational change in MreB and thus stabilize a less-twisted double protofilament. Further studies are needed to completely dissect the energetic landscape of MreB conformations.

Much remains to be learned about the links among MreB, its regulatory partners, and cell-wall synthesis. Here, our simulations predict that RodZ binding can reduce the intrinsic twist of MreB and may even reverse chirality (Fig. 4c), highlighting the need for further studies that quantitatively interrogate MreB filament conformation in vivo. In addition to limit length and orientation, cellular factors that interact with MreB could also affect the size distribution of MreB filaments by modulating MreB polymerization kinetics. Further studies are needed to quantify such effects. The curvature-inducing ability of MreB (Fig. 3g–i) may be important for the geometric localization of MreB^8^. While in vitro assays of the interaction of MreB with the membrane are challenging due to MreB’s N-terminal amphiphilic helix, further coarse-grained approaches incorporating the mechanical properties of the membrane and turgor pressure will further broaden our understanding of MreB’s role in geometric sensing and cell-shape determination. Although previous models have studied how MreB orientation is related to filament mechanics^23,35^, they have either assumed a non-twisted filament conformation^23^, or neglected the fact that membrane binding only occurs on a specific side of the filament^35^. Another related theoretical model for FtsZ filaments incorporated both twisting and membrane binding, but was limited to long filaments on flat membrane surfaces^36^. Our course-grained model suggests that the interplay between twist and binding may determine the filament lengths and orientation, thus providing an expanded view of MreB mechanics and ultrastructure.

Beyond MreB, many other bacterial actin homologs such as FtsA, ParM, and MamK also polymerize into filaments. While these proteins have diverse roles in bacteria, our study suggests that nucleotide binding and protein–protein interactions may generally induce conformational changes in these polymers whose discovery can be accelerated with MD simulations^37^. Despite their common structural homology to actin, these proteins exhibit diverse protofilament architectures^38^, which may reflect their varied physiological roles from cell division to plasmid segregation. That binding of RodZ or genetic mutations in MreB may alter or even reverse chirality (Fig. 4b, c) reflects remarkable flexibility in the intrafilament interface of MreB, wherein single mutations can exert enormous impact on mesoscopic filament conformation and cell shape. Chirality reversal in mammalian cells distinguishes cancerous cells from normal cells, and such chirality is dependent on the functionality of the actin cytoskeleton^39^. Moreover, modulation of chirality is not limited to the actin family: single mutations can also introduce twist to filaments of the bacterial tubulin homolog FtsZ, resulting in growth along a helical pattern rather than a ring^40^. Thus, future efforts to understand the molecular origin of chirality in cytoskeletal filaments should have broad implications for studying chiral morphogenesis and identifying potential factors that alter or reverse chirality.

## Methods

### Modeling the amphipathic helix of MreB

The amphipathic helix in CcMreB (residues 1–8) was not resolved in the crystal structure, but plays an important role in membrane binding^5^. We modeled the amphipathic helix using the Molefacture Protein Builder plugin in VMD by aligning the residues into an alpha helix (peptide backbone torsion angles phi = −57°, psi = −47°), and combining the resulting helix with the main crystal structure.

### Equilibrium MD simulations

All simulations except the 500-ns simulations in Fig. 3c were performed using the MD package NAMD^41^; the 500-ns simulations in Fig. 3c were performed on the Anton 2 supercomputer^42^. All simulations used the CHARMM36 force field^43^, including CMAP corrections^44^. Water molecules were described with the TIP3P model^45^. Long-range electrostatic forces were evaluated by means of the particle-mesh Ewald summation approach with a grid spacing of <1 Å. An integration time step of 2 fs was used^46^. Bonded terms and short-range, nonbonded terms were evaluated every time step, and long-range electrostatics were evaluated every other time step. Constant temperature (*T* = 310 K) was maintained using Langevin dynamics^47^, with a damping coefficient of 1.0 ps^−1^. A constant pressure of 1 atm was enforced using the Langevin piston algorithm^48^ with a decay period of 200 fs and a time constant of 50 fs. Setup, analysis, and rendering of the simulation systems were performed with the software VMD^49^. Steering of the opening angle was achieved by introducing collective forces to constrain the angle to defined values through the collective variable functionality of NAMD^41^.

### Simulated systems

MD simulations performed in this study are described in Supplementary Table 1. Unless otherwise noted, systems were initialized from the crystallographic structure of *C. crescentus* MreB bound to magnesium and ADP (PDB ID: 4CZF)^5^. The bound nucleotide was replaced by ATP or ADP with chelating Mg^2+^ ions for all simulated systems. In simulations including a membrane, patches consisting of phosphatidylethanolamine were generated using the membrane plugin in VMD. Water and neutralizing ions (Na^+^ or Cl^−^) were added around each simulated system, resulting in final simulation sizes of up to 480,000 atoms. For mean values and distributions of measurements, only the last 40 ns were used for each simulation. All simulations were run until equilibrium was reached unless specified in the text. To ensure simulations had reached equilibrium, measurement distributions were fit to a Gaussian.

### Analysis of dihedral and opening angles

The centers-of-mass of the four subdomains of each protein subunit were obtained using VMD, excluding the amphiphilic helix (residues 1–8). For each time step, we calculated one opening angle from the dot product between the vector defined by the centers-of-mass of subdomains IIA and IIB and the vector defined by the centers-of-mass of subdomains IIA and IA. Similarly, we calculated a second opening angle from the dot products between the vectors defined by the centers-of-mass of subdomains IA and IB and of subdomains IA and IIA. The opening angles we report are the average of these two opening angles (Fig. 2b, left). The dihedral angle was defined as the angle between the vector normal to a plane defined by subdomains IA, IB, and IIA and the vector normal to a plane defined by subdomains IIB, IIA, and IA (Fig. 2b, right).

### Calculation of bending and twist angles in protofilaments

At each time step of a simulation, the coordinate system of the bottom and top subunits (or each subunit pair) was defined using three unit vectors (**d**_**1**_–**d**_**3**_)^50^. For single protofilaments, **d**_**3**_ approximately aligns to the center of mass between the two subunits, **d**_**2**_ is defined to be perpendicular to the membrane plane, and **d**_**1**_ = **d**_**3**_ × **d**_**2**_ (Fig. 2d–g). The same definitions for the unit vectors were used for double protofilaments. The rotation angle around **d**_**3**_ (*θ*_3_) represents twist between the bottom and top subunits (or subunit pairs). Similarly, rotations around **d**_**2**_ and **d**_**1**_ (*θ*_2_ and *θ*_1_) represent bending parallel to the membrane plane and bending toward the membrane plane, respectively (Fig. 2d–g).

### A22 force field generation

The A22 structure was isolated from PDB ID 4CZG using UCSF chimera^51^ by removing all other molecules and adding missing hydrogens in the original PDB file. The force field file for A22 was generated using SwissParam with default parameters^52^.

### Calculation of buried SASA

The interaction strength between two interacting molecules was estimated by calculating the contact surface area between them, which can be approximated by measuring the surface area buried between the two molecules that is not accessible to solvent when the molecules interact. This surface area is known as the buried SASA. The buried SASA between two molecules can be calculated from three quantities: the SASA of each molecule by itself (denoted as *A*_1_ and *A*_2_), and the SASA of the complex of the two molecules when interacting (denoted as *A*_1+2_). If the molecules are in contact, then the sum of the SASA of each molecule is greater than the SASA for both molecules together, and the contact area is the difference between the two values divided by two (to account for double counting):

buried SASA = (*A*_1_ + *A*_2_ − *A*_1+2_)/2.

For each molecule, its corresponding SASA was calculated in VMD using the command ‘measure sasa’, with 1.40 Å as the van der Waal’s radius for water molecules.

### Construction of protein homology models

Homology models were constructed using the software MODELER^53^. Using MreB as an example, the amino acid sequences of EcMreB and CcMreB were aligned using the UniProt website (http://www.uniprot.org/align/). The alignment results and the PDB file with the CcMreB crystal structure were processed by MODELLER to generate 10 homology models. The homology model with the lowest DOPE-HR score was used for MD simulations.

### Calculation of membrane patch curvature in simulations

The positions of each phosphate atom in the top layer of the membrane (the layer that directly interacts with MreB) were extracted and fit to a second-order polynomial. The curvature of the membrane patch was defined as the curvature at the center of the fitted surface.

### Coarse-grained analytical model

The Hamiltonian of the filament is^30^

1 $$H = \frac{1}{2}\int_{-L/2}^{L/2} {{\mathrm{d}}s\left[ {C\left( {\frac{{{\mathrm{sin}}^2\theta }}{r} - k_0} \right)^2 + C\left( {\theta^{\prime}} \right)^2 + K\left( {\psi \prime - \frac{{{\mathrm{sin}}2\theta }}{{2r}} - \omega _0} \right)^2 + 2V{\mathrm{sin}}^2\left( {\frac{\psi }{2}} \right)} \right]} ,$$

where *L* is the total length of the filament, *θ* and *ψ* are the local tilt and twist angles, respectively, *θ*’ and *ψ*’ are the corresponding derivatives with respect to filament length coordinate *s*, *r* is the radius of the cell, *C* is the bending modulus of the filament, *K* is the torsional modulus, *V* is the membrane binding potential, and *k*_0_ and *ω*_0_ are the intrinsic curvature and twist of the filament, respectively. Note that in the original model in Ref. ^30^, the membrane-binding interface was assumed to lie on two opposing sides of the filament (both the top and the bottom side), such that maximum membrane binding was achieved by both *ψ* = 0 and *ψ* = 180°. However, in the case of MreB, filaments only have one side that can bind to the membrane^5^. Therefore, we adjusted the model so that maximum membrane binding can only occur when *ψ* = 0, and no membrane-binding would occur with *ψ* = 180°, by modifying the membrane-binding term from $V{\mathrm{sin}}^2\left( \psi \right)$ to $V{\mathrm{sin}}^2\left( {\frac{\psi }{2}} \right)$. Parameter values are listed in Supplementary Table 2.

We analytically minimized the Hamiltonian in Eq. 1 under the assumption that the membrane is flat, i.e., *r* → *∞*. In this case

2 $$H = \frac{1}{2}\int_{ - L/2}^{L/2} {{\mathrm{d}}s\left[ {Ck_0^2 + K\left( {\psi^{\prime} - \omega _0} \right)^2 + 2V{\mathrm{sin}}^2\left( {\frac{\psi }{2}} \right)} \right]} .$$

When *H* is minimized, $\frac{{\delta H}}{{\delta \psi }} - \frac{\partial }{{\partial s}}\frac{{\delta H}}{{\delta \psi ^{\prime} }} = 0$, which gives

3 $$K\psi^{\prime\prime} = V\sin \left( {\frac{\psi }{2}} \right)\cos \left( {\frac{\psi }{2}} \right)$$

and is equivalent to

4 $$\frac{\mathrm{d}}{{\mathrm{d}}s}\left[ {\frac{K}{2}\left( {\psi^{\prime} } \right)^2 - V{\mathrm{sin}}^2\left( {\frac{\psi }{2}} \right)} \right] = 0.$$

Integrating Eq. 4 and utilizing the boundary conditions $\psi^{\prime} |_{s = \pm L/2} = \omega _0$ and $\psi |_{s = \pm L/2} = \pm \psi _m$, we obtain

5 $$\frac{{\mathrm{d}\psi }}{{\mathrm{d}}s} = \sqrt {\omega _0^2 + \frac{{2V}}{K}\left( {{\mathrm{sin}}^2\left( {\frac{\psi }{2}} \right) - {\mathrm{sin}}^2\left( {\frac{{\psi _m}}{2}} \right)} \right)} .$$

Integration of Eq. 5 from -*L*/2 to *L*/2 gives

6 $$L\left( {\psi _m} \right) = \int_{- \psi _m}^{\psi _m + 2n\pi } {\frac{{\mathrm{d}\psi }}{{\sqrt {\omega _0^2 + \frac{{2V}}{K}\left( {{\mathrm{sin}}^2\left( {\frac{\psi }{2}} \right) - {\mathrm{sin}}^2\left( {\frac{{\psi _m}}{2}} \right)} \right)} }}} .$$

The total energy of the filament can be similarly calculated as

7 $$E\left( {\psi _m} \right) =	 \, K\int_{ - \psi _m}^{\psi _m + 2n\pi } {\mathrm{d}}\psi \sqrt {\omega _0^2 + \frac{{2V}}{K}\left( {{\mathrm{sin}}^2\left( {\frac{\psi }{2}} \right) - {\mathrm{sin}}^2\left( {\frac{{\psi _m}}{2}} \right)} \right)} - 2K\omega _0\left( {\psi _m + n{\it{\uppi }}} \right)\\ \, 	+ V{\mathrm{sin}}^2\left( {\frac{{\psi _m}}{2}} \right)L + e_0 L ,$$

where $e_0 = \frac{C}{2}k_0^2$ and *n* is the number of full twist repeats in the filament.

Integrating the above equations, we obtained the final expressions for *L* and *E*:

8 $$L\left( {\psi _m} \right) =	 \, \frac{{4\sqrt 2 }}{{\omega _0}}\sqrt {\frac{{l_m^2}}{{2l_m^2 + \cos \psi _m - 1}}} F\left[ {\frac{{\psi _m}}{2}, - \frac{2}{{2l_m^2 + \cos \psi _m - 1}}} \right]\\ \, 	+ \frac{{8\sqrt 2 n}}{{\omega _0}}\sqrt {\frac{{l_m^2}}{{2l_m^2 + \cos \psi _m - 1}}} K\left[ { - \frac{2}{{2l_m^2 + \cos \psi _m - 1}}} \right],$$

where $F\left[ {\psi ,k} \right]$ is the incomplete elliptical integral of the first kind, $K\left[ k \right]$ is the complete elliptical integral of the first kind, and $l_m = \sqrt {\frac{K}{{2V}}} \omega _0$, while

9 $$E\left( {\psi _m} \right) =	 \, 2\sqrt 2 K\omega _0\sqrt {\frac{{2l_m^2 + \cos \psi _m - 1}}{{l_m^2}}} E\left[ {\frac{{\psi _m}}{2}, - \frac{2}{{2l_m^2 + \cos \psi _m - 1}}} \right]\\ \, 	+ 4\sqrt 2 nK\omega _0\sqrt {\frac{{2l_m^2 + \cos \psi _m - 1}}{{l_m^2}}} E\left[ { - \frac{2}{{2l_m^2 + \cos \psi _m - 1}}} \right]\\ \, 	- 2K\omega _0\left( {\psi _m + n{\it{\uppi }}} \right) + V{\mathrm{sin}}^2\left( {\frac{{\psi _m}}{2}} \right)L + e_0L,$$

where $E\left[ {\psi ,k} \right]$ and $E\left[ k \right]$ are the incomplete and complete, respectively, elliptical integral of the second kind.

With the analytical solutions of *L* and *E* in Eqs. 8 and 9, respectively, we can numerically determine the relationship between them by varying *ψ*_*m*_ and *θ*_0_. Since MreB filaments are unlikely to exhibit a full 360° twist without breaking, we only considered the case *n* = 0. The limit length of the filament is dictated by $\frac{{\mathrm{d}}E}{{\mathrm{d}}L} = \mu _0$, where *μ*_0_ is the MreB polymerization energy per unit length.

### Monte Carlo simulations

For a filament of length *L*, we minimized its Hamiltonian in Eq. 1 with respect to *θ* and *ψ* to obtain the equilibrium filament shape. The energy was computed by discretizing the Hamiltonian into *N* segments, with each segment *i* able to adopt a distinct bending and twisting conformation described by angles *θ*_*i*_ and *ψ*_*i*_. The discretized Hamiltonian was used to calculate the total energy of the filament as

10 $$E = 	\, \frac{1}{2} \sum_{i = 1}^N \left[ C\left( {\frac{{{\mathrm{sin}}^2\overline {\theta _{i,i + 1}} }}{r} - k_0} \right)^2 + C\left( {\Delta \theta _{i,i + 1}} \right)^2 + K\left( {\Delta \psi _{i,i + 1} - \frac{{{\mathrm{sin}}2\overline {\theta _{i,i + 1}} }}{{2r}} - \omega _0} \right)^2\right. \\ 	+ 2V{\mathrm{sin}}^2\left( {\frac{{\overline {\psi _{i,i + 1}} }}{2}} \right) \Bigg] ,$$

where $\overline {\theta _{i,i + 1}}$ and $\overline {\psi _{i,i + 1}}$ are the average tilt and twist angles between nearest neighbor segments, and $\Delta \theta _{i,i + 1}$ and $\Delta \psi _{i,i + 1}$ are the differences in tilt and twist angles between nearest neighbor segments. A classical Metropolis Monte Carlo algorithm was used to minimize the energy of the system. Specifically, starting from an initial configuration of *θ* = 90° and *ψ* = 0, each Monte Carlo step *t* altered *θ*_*i*_ or *ψ*_*i*_ to change the filament conformation from *z*_*t*_ to a trial conformation *z’*. The new conformation *z*_*t*+1_ was determined using the Metropolis algorithm

11 $$z_{t + 1} = \left\{ {\begin{array}{*{20}{c}} {z^\prime\; {\rm{with}}\,{\rm{probability}}\,p = {\mathrm{e}}^{ - \left( {{\mathrm{E}}\left( {z^\prime } \right) - E\left( {z_t} \right)} \right)/k_BT}\, or\; z_t\; {\rm{with}}\; {\rm{probability}}\,1 - p,{\rm{if}}\,E\left( {z^{\prime} } \right) > E\left( {z_t} \right)} \\ {z^{\prime} ,{\rm{if}}\,E\left( {z^{\prime} } \right) \le E\left( {z_t} \right)} \end{array}} \right.$$

Results were assessed to have converged after ~10^7^ Monte Carlo steps if the energy fluctuations were lower than 1% of the minimized energy across the previous 10^4^ steps. Twenty independent replicate simulations were carried out for each parameter set to ensure that a global minimum was reached.

### Estimation of parameters for coarse-grained modeling

The bending and torsional moduli of MreB filaments were estimated from the variance of the appropriate MD simulations. For the torsional modulus *K*, the standard deviation, *σ*, of the fluctuations in the twist angle from 4 × 2 protofilament simulations was ~1.88° per monomer length. From this value, the torsional rigidity can be estimated as *K* = *k*_B_*T*∆*l*/*σ*^2^, where ∆*l* ~5 nm is the length of an MreB monomer. This estimate gives *K* ~4.6 × 10^3^ *k*_B_*T* nm. The bending modulus *C* can be estimated similarly. Simulations of MreB filaments revealed bending in two directions dictated by $\theta _1$ and $\theta _2$ (Fig. 2d–g), but the corresponding bending moduli *C*_1_ and *C*_2_ were very similar (<5% differences, Supplementary Fig. 2a, b), and therefore we used their average *C* as the bending modulus for bending in both directions in our coarse-grained simulations. Mutations in MreB did not lead to substantially different estimates of *K* or *C* (Supplementary Fig. 3a–c). The intrinsic twist rate, *ω*_0_, was determined from the twist angle $\theta _3$, and varied across MreB mutants. Similarly, the intrinsic curvature of the filament, *k*_0_, was determined from $\theta _1$ and $\theta _2$. As $\theta _2$ remained near zero, *k*_0_ was largely dictated by $\theta _1$. For all mutants, *k*_0_ = 2.3 × 10^−3^ rad/nm. Similarly, the membrane-binding potential of each MreB monomer was estimated from the buried SASA dynamics (Supplementary Fig. 3j) to be 10 *k*_B_*T*, yielding *V* = 4 *k*_B_*T* nm^−1^ for a double protofilament. The polymerization free energy was estimated as $\Delta G = - RT{\mathrm{ln}}\left( {K_{\mathrm{c}}} \right)$, where *K*_c_ is the equilibrium constant, defined as the ratio between off and on rates of polymerization. Using parameters for actin^29^, ∆*G* for an MreB monomer is ~5 *k*_B_*T*, equivalent to *µ*_0_ = 2 *k*_B_*T* nm^−1^ for a double protofilament. See Supplementary Table 2 for a list of all parameters used in our coarse-grained simulations.

### Strains and media

Strains used in this study are listed in Supplementary Table 3. All strains were grown with aeration at 37 °C in LB medium (10 g/L tryptone, 5 g/L yeast extract, and 5 g/L NaCl).

### Structured illumination microscopy

Saturated overnight cultures were back-diluted 1:200 into prewarmed fresh LB and grown at 37 °C with shaking. The cultures were further diluted 1:10 into prewarmed fresh LB at 60 and 150 min after the first dilution, respectively. By 220 min, the cultures reached exponential growth with OD ~0.1. One milliliter of the cells was fixed in phosphate-buffered saline containing 3% glutaraldehyde/3% paraformaldehyde (electron microscopy sciences) at room temperature for 15 min, with 1 μg/mL FM 4-64FX membrane stain (Invitrogen) added during fixation. Cells were washed three times in cold phosphate-buffered saline, and 1 µL of the cell solution was pipetted onto a No. 1.5 coverslip (Zeiss) coated with poly-l-lysine solution (Sigma-Aldrich). After the droplet dried, a small drop of ProLong Diamond AntiFade Mountant (Thermo Fisher) was added on top of the dried droplet, and the coverslip was mounted on a glass slide (VWR) and sealed with VALAP (equal parts Vaseline, lanolin, and paraffin by weight).

Cell samples were imaged on an OMX V4 microscope platform (GE Life Sciences) with a 100× (NA 1.42) oil-immersion objective (Nikon Instruments). Images from two channels were collected on two Evolve 512 electron-multiplying charged couple device cameras (Photometrics) using DeltaVision microscopy imaging system v. 3.70 (GE Life Sciences).

### Image analyses of structured illumination microscopy

Raw images were reconstructed and aligned into 3D *z*-stacks using SoftWoRx v. 6.5.2 (GE Life Sciences). The middle plane for each *z*-stack was segmented using the FM 4-64FX signal using Morphometrics^54^ to obtain individual cell contours. For each contour, a coordinate-system mesh was calculated using the pill mesh function from MicrobeTracker^55^. A three-dimensional surface was reconstructed from the segmentation mesh assuming rotational symmetry about the central axis, and MreB patches localized near the cell periphery were identified from the GFP channel based on intensity using the MATLAB function im2bw with default parameters. Patches smaller than the resolution limit for structured illumination microscopy (~0.02 μm^2^) were excluded from further quantification. Since all the patches were largely shaped as a band, the length of each patch’s major axis was defined as the filament length, and the pitch angle was defined as the orientation of the major axis.

## Supplementary information

Supplementary Information

Description of Additional Supplementary Information

Supplementary Movie 1

Supplementary Movie 2

## Data Availability

Source data for the figures are provided as a Source Data file. Other datasets generated during the current study are available from the corresponding author on request.

## Source data

Source Data

## Code availability

The scripts for generating and plotting the Monte Carlo results are deposited in https://bitbucket.org/kchuanglab/mc_finite_filament/src/master/.
