# Supplementary material for: Chiral twisting in a bacterial cytoskeletal polymer affects filament size and orientation
Source: Nat Commun. 2020 Mar 16;11:1408. doi: 10.1038/s41467-020-14752-9 (PMC7075873; doi:10.1038/s41467-020-14752-9)
Supplement: Supplementary file 1 — Supplementary Information [file 41467_2020_14752_MOESM1_ESM.pdf]

Supplementary Information

Chiral twisting in a bacterial cytoskeletal polymer affects filament size and orientation

Shi *et al.*

Supplemental Tables

Supplementary Table 1: List of MD simulation systems from this study.

Name	Structure source	Ligand	Atoms (x1000)	Time (ns)	Replicates
1x1 ATP	PDB: 4CZF	ATP and Mg^{2+}	95	80	2
1x1 ADP	PDB: 4CZF	ADP and Mg^{2+}	95	80	2
1x1 ATP A22	PDB: 4CZF	ATP, A22, and Mg^{2+}	95	70	1
1x1 ATP steered	PDB: 4CZF	ATP and Mg^{2+}	95	70	2
1x1 ATP (TmMreB)	PDB: 1JCG	ATP and Mg^{2+}	95	80	2
2x1 ATP	PDB: 4CZF	2xATP and $2xMg^{2+}$	130	120	2
2x1 ADP	PDB: 4CZF	2xADP and $2xMg^{2+}$	130	120	2
4x2 ATP	PDB: 4CZF	8xATP and $8xMg^{2+}$	356	500	2
4x2 ADP	PDB: 4CZF	8xADP and $8xMg^{2+}$	356	500	2
8x2 ATP	PDB: 4CZF	16xATP and $16xMg^{2+}$	689	57	1
4x2 ATP (EcMreB)	PDB: 4CZF homology model	8xATP and $8xMg^{2+}$	356	100	2
4x2 ATP membrane	PDB: 4CZF	8xATP and $8xMg^{2+}$, membrane patch	434	120	2

4x2 ADP membrane	PDB: 4CZF	8xADP and 8xMg^{2+}, membrane patch	434	120	2
2x1 ATP membrane	PDB: 4CZF	2xATP and 2xMg^{2+}, membrane patch	202	80	1
2x1 ADP membrane	PDB: 4CZF	2xADP and 2xMg^{2+}, membrane patch	202	80	1
4x1 ATP membrane	PDB: 4CZF	4xATP and 4xMg^{2+}, membrane patch	437	80	2
4x2 ATP (E275D)	PDB: 4CZF (E275D mutation)	8xATP and 8xMg^{2+}	356	120	2
4x2 ATP (I138V)	PDB: 4CZF (I138V mutation)	8xATP and 8xMg^{2+}	356	120	2
4x2 ATP (R121C)	PDB: 4CZF (R121C mutation)	8xATP and 8xMg^{2+}	356	120	2
4x2 ATP (V53A)	PDB: 4CZF (V53A mutation)	8xATP and 8xMg^{2+}	356	120	2
4x2 ATP (EcMreB, E276D)	PDB: 4CZF homology model	8xATP and 8xMg^{2+}	356	100	2

	(E276D mutation)				
4x2 ATP (EcMreB, I141V)	PDB: 4CZF homology model (I141V mutation)	8xATP and 8xMg^{2+}	356	100	2
4x2 ATP (EcMreB, R124C)	PDB: 4CZF homology model (R124C mutation)	8xATP and 8xMg^{2+}	356	100	2
4x2 ATP (EcMreB, V55A)	PDB: 4CZF homology model (V55A mutation)	8xATP and 8xMg^{2+}	356	100	2
1x1 ATP membrane	PDB: 4CZF	ATP and Mg^{2+}, membrane patch	128	100	2
1x1 ATP membrane (E275D)	PDB: 4CZF (E275D mutation)	ATP and Mg^{2+}, membrane patch	128	100	1

1x1 ATP membrane (I138V)	PDB: 4CZF (I138V mutation)	ATP and Mg^{2+}, membrane patch	128	100	1
1x1 ATP membrane (R121C)	PDB: 4CZF (R121C mutation)	ATP and Mg^{2+}, membrane patch	128	100	1
1x1 ATP membrane (V53A)	PDB: 4CZF (V53A mutation)	ATP and Mg^{2+}, membrane patch	128	100	1
4x2 ATP membrane (R121C)	PDB: 4CZF (R121C mutation)	8xATP and 8xMg^{2+}, membrane patch	434	120	2
4x2 ATP membrane (V53A)	PDB: 4CZF (V53A mutation)	8xATP and 8xMg^{2+}, membrane patch	434	120	2
4x2 ATP RodZ	PDB: 4CZF (for MreB) PDB: 2WUS (for RodZ, homology model)	8xATP and 8xMg^{2+}	481	100	2

4x2 ATP (V53A) RodZ	PDB: 4CZF (for MreB, with V53A mutation) PDB: 2WUS (for RodZ, homology model)	8xATP and 8xMg^{2+}	481	100	2

Supplementary Table 2: Parameter values for the coarse-grained model.

Parameter	Physical meaning	Value
C	Bending modulus of MreB filament	1.4×10^4 $k_B T$ nm
K	Torsional rigidity	4.6×10^3 $k_B T$ nm
k_0	Intrinsic curvature	2.3×10^{-3} rad nm^{-1}
ω_0	Intrinsic twisting rate	0.01 – 0.05 rad nm^{-1}<br>0.033 rad nm^{-1} for wild-type
r	Cell radius	400 nm
V	Membrane binding potential	4 $k_B T$ nm^{-1}
μ_0	Polymerization free energy	2 $k_B T$ nm^{-1}

Supplementary Table 3: Strains used in this study.

Strain name	Genotype	Source
KC508	MG1655 *csrD::Km, mreB'::msfGFP-mreB''*	[1]
KC507	MG1655 *csrD::Km, mreB'::msfGFP-mreB''-E276D*	[1]
KC968	MG1655 *csrD::Km, mreB'::msfGFP-mreB''-R124C*	[2]
MreB(msfGFP)-EP028	MG1655 Δ*mreB* [pRMmreBCD-V55A-msfGFP]	[3]
MreB(msfGFP)-EP067	MG1655 Δ*mreB* [pRMmreBCD-I141V-msfGFP]	[3]

Supplemental Figures

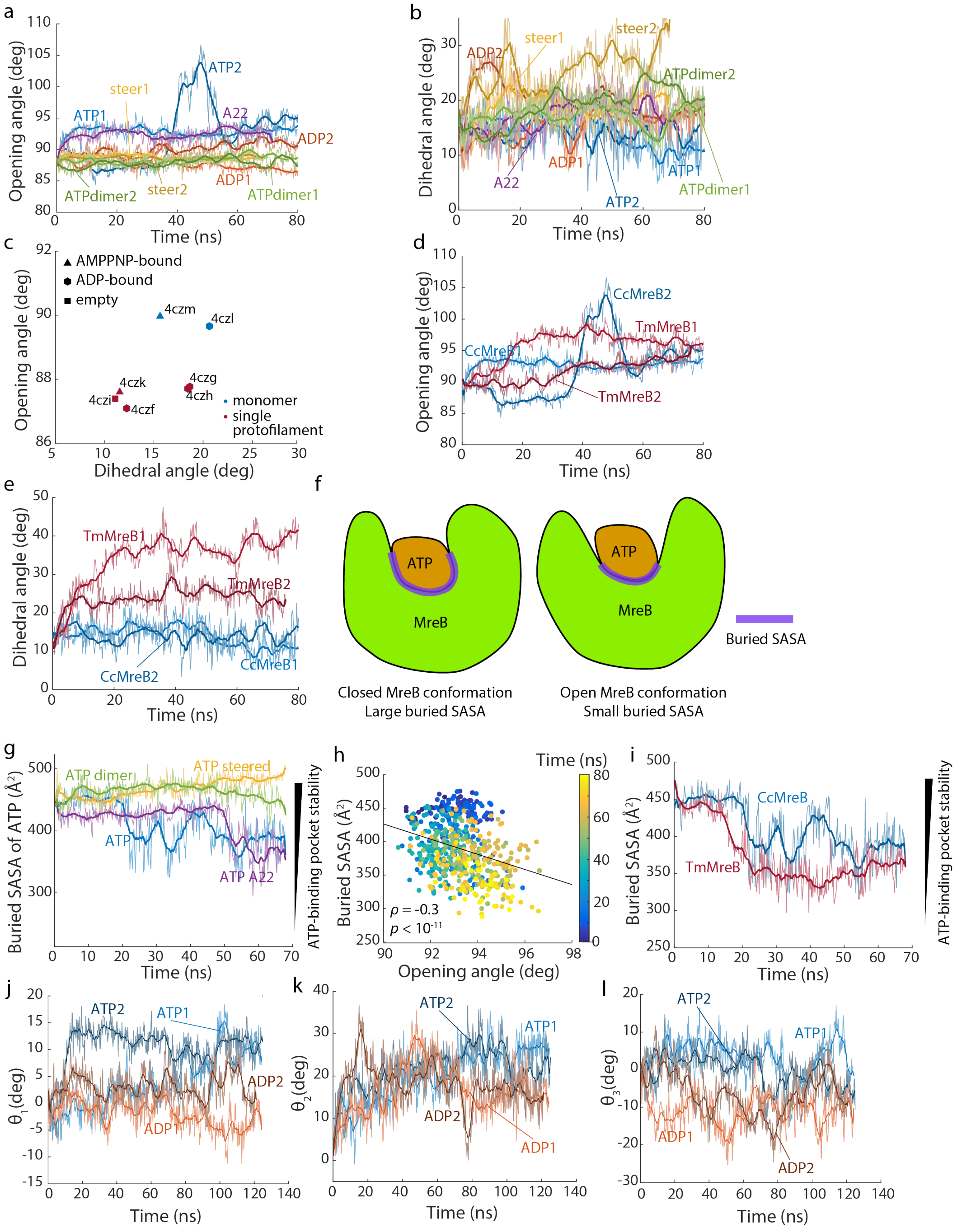

Supplementary Figure 1: MreB monomer and dimer conformations are nucleotide-dependent.

a-b) Trajectories of opening angles (a) and dihedral angles (b) in each simulated system. For dimer simulations, the calculated values are for the (-) subunit. Thick

lines are the results of smoothing the raw data (light lines) using a sliding window of 20 frames. ATP-bound monomers had larger opening angles than other systems.

c) Scatter plots of opening and dihedral angles in CcMreB crystal structures. The monomeric crystal structures (blue) have larger opening angles than those forming single protofilaments (red).

d-e) Trajectories of (d) opening angles and (e) dihedral angles in ATP-bound CcMreB and TmMreB. TmMreB exhibited larger dihedral angles than CcMreB; results were more variable for TmMreB than CcMreB across two replicate simulations. Thick lines are the results of smoothing the raw data (light lines) using a sliding window of 20 frames.

f) Schematic of buried solvent-accessible surface area (SASA) between ATP and MreB. A closed MreB conformation has a larger interaction region with ATP (purple shading), and therefore a larger buried SASA than an open MreB conformation.

g) Trajectories for buried SASA of ATP. Buried SASA is related to the open or closed states of the MreB monomer. Open states (ATP- or ATP-A22-bound) had lower buried SASA values, whereas closed states (dimer or steered simulations) maintained high buried SASA values, indicating a stabilized ATP-binding pocket that may facilitate ATP hydrolysis. Thick lines are the results of smoothing the raw data (light lines) using a sliding window of 20 frames.

h) Scatter plots of buried SASA and opening angle in a simulation of an ATP-bound MreB monomer. Buried SASA negatively correlated with opening angle

(Spearman's ρ = -0.3, $p < 10^{-11}$ using permutation test), suggesting that an open conformation destabilizes the ATP-binding pocket. Each dot represents one time point, with the dots colored by simulation time.

i) Trajectories of buried SASA in ATP-bound CcMreB and ATP-bound TmMreB. In both systems, the buried SASA dropped similarly, indicating that although TmMreB and CcMreB conformations behaved differently, their interactions with ATP were similar. Thick lines are the results of smoothing of the raw data (light lines) using a sliding window of 20 frames.

j-l) Trajectories of the three Euler angles in each simulated MreB dimer system. ATP-bound dimers consistently exhibited larger θ_1 (j) and θ_2 (k) values than ADP-bound dimers; no clear twisting in θ_3 was observed (l). Thick lines are the results of smoothing the raw data (light lines) using a sliding window of 20 frames.

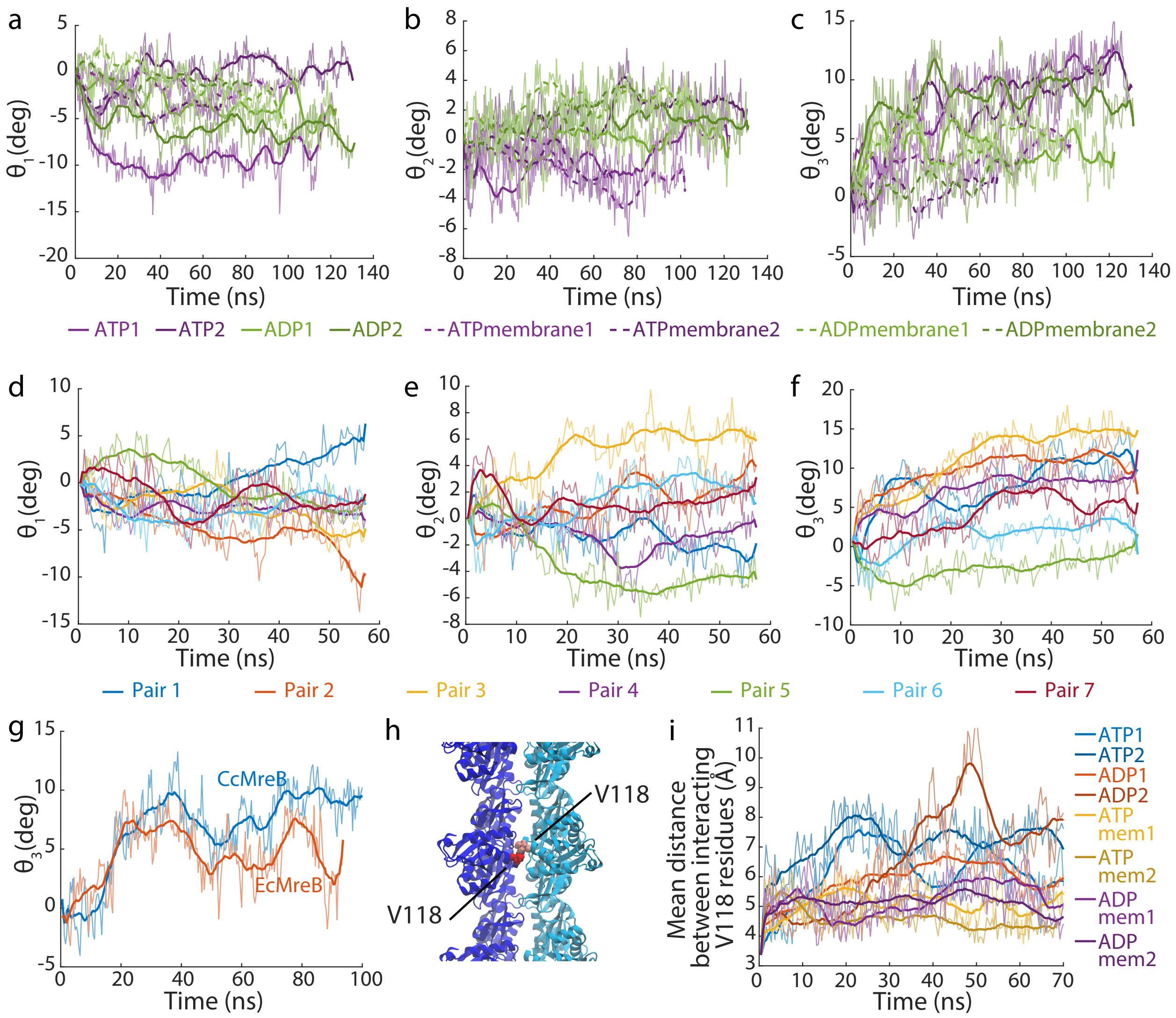

Supplementary Figure 2: Interaction between a CcMreB double protofilament and a membrane.

a-c) Trajectories of the three Euler angles in each simulated system of MreB 4x2 protofilaments, calculated for the middle doublet pair (Pair 2, Fig. 3a). θ_1 and θ_2 remained close to zero, whereas the ATP-bound protofilaments in water exhibited larger twisting in θ_3. Thick lines are the results of smoothing the raw data (light lines) using a sliding window of 20 frames.

d-f) Trajectories of the three Euler angles in each neighboring pair of MreB doublets in an 8x2 protofilament simulation. Although the system still exhibited large variability by the end of the simulation, θ_1 and θ_2 largely remained close to zero, while the values of θ_3 were comparable to those in ATP-bound 4x2 simulations (panels a-c). Thick lines are the results of smoothing the raw data (light lines) using a sliding window of 20 frames.

g) Trajectories of twisting angle θ_3 for 4x2 simulations of CcMreB and EcMreB. EcMreB exhibited quantitatively similar twisting angles as CcMreB, suggesting that our simulation results in CcMreB are applicable to *E. coli*. Thick lines are the results of smoothing the raw data (light lines) using a sliding window of 20 frames.

h) V118 residues facilitate inter-protofilament interaction between two antiparallel MreB protofilaments.

i) The mean distance between interacting V118 residues increased over time, suggesting a loss of inter-protofilament interaction. For membrane-bound simulations, the V118 distances remained small, indicating that the membrane stabilizes the double protofilament conformation. Thick lines are the results of smoothing of the raw data (light lines) using a sliding window of 20 frames.

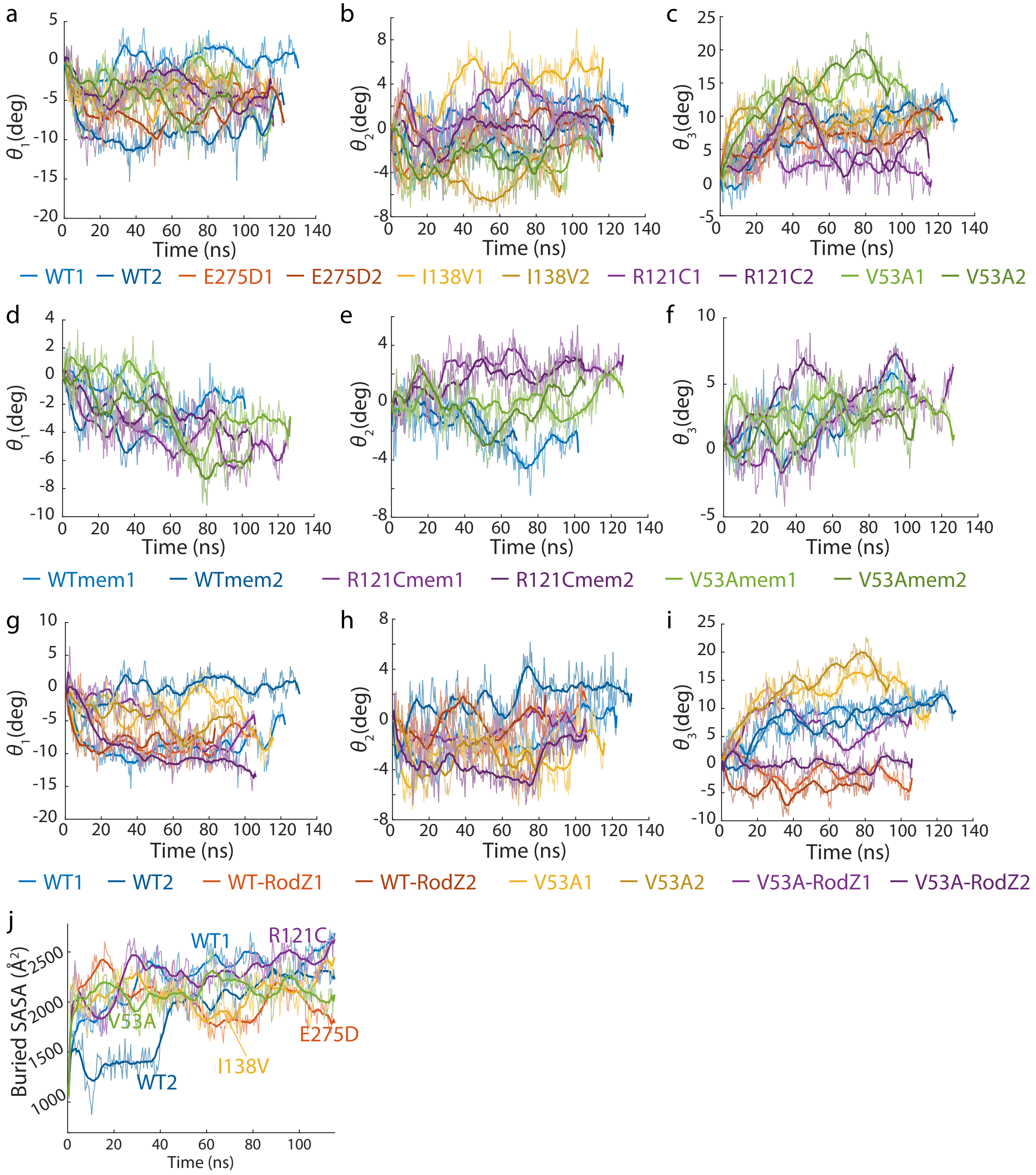

Supplementary Figure 3: MreB twist angle is affected by mutations and RodZ binding.

a-c) Trajectories of the three Euler angles in 4x2 protofilaments of MreB mutants, calculated for the middle doublet pair (Pair 2, Fig. 3a). θ_1 and θ_2 were unaffected,

while θ_3 was systematically tuned by mutations. Thick lines are the results of smoothing the raw data (light lines) using a sliding window of 20 frames.

d-f) Trajectories of the three Euler angles in 4x2 protofilaments of wild-type MreB and R121C and V53A mutants bound to a membrane patch, calculated for the middle doublet pair (Pair 2, Fig. 3a). In both mutants, all three angles behaved similarly as in wildtype. Despite the distinct values of θ_3 in water, membrane binding suppressed all twisting to similar extents. Thick lines are the results of smoothing the raw data (light lines) using a sliding window of 20 frames.

g-i) Trajectories of the three Euler angles in 4x2 protofilaments of wild-type MreB and the V53A mutant, with or without the cytoplasmic tail of RodZ. θ_1 and θ_2 were largely unaffected, while θ_3 decreased upon RodZ binding in both wildtype and the V53A mutant. Thick lines are the results of smoothing the raw data (light lines) using a sliding window of 20 frames.

j) Buried SASA of the membrane-binding interface for CcMreB wild-type and mutant monomers. All simulations exhibited similar buried SASA at equilibrium, suggesting that the mutations did not alter the membrane binding properties of MreB.

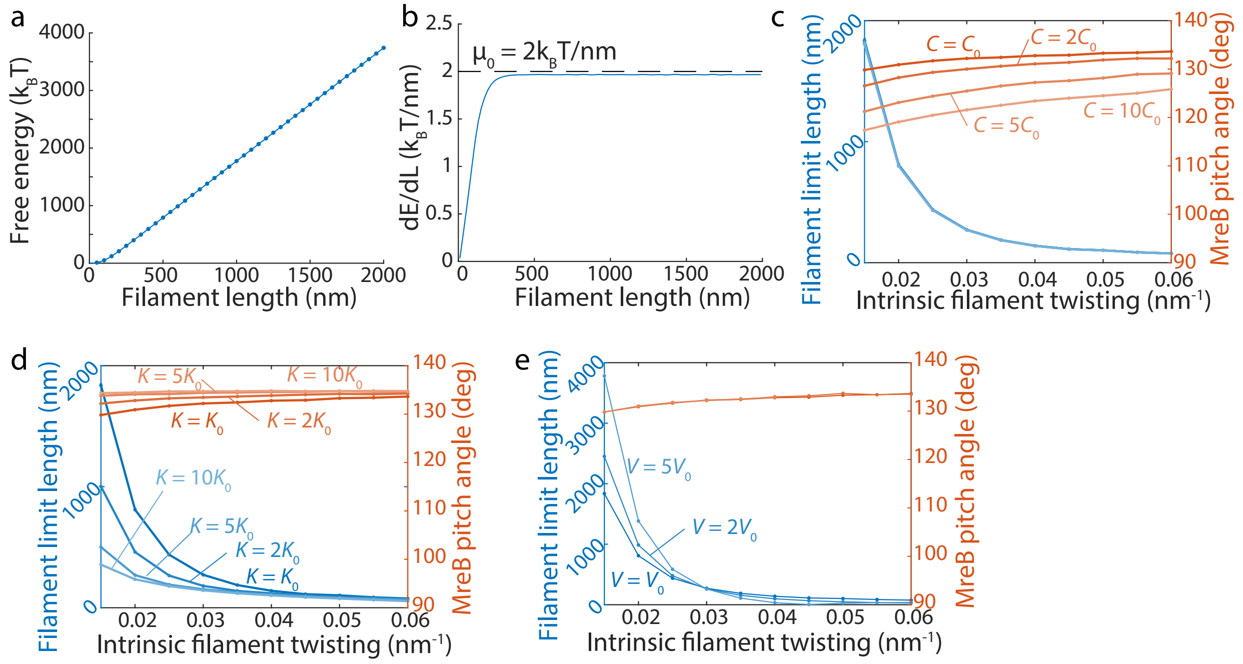

Supplementary Figure 4: MreB twist angle predicts MreB filament limit length and orientation *in vivo*.

a) Filament free energy as a function of filament length when bound to a cylindrical membrane with radius r = 80 nm.

b) For the filament in (a), $\frac{dE}{dL} < \mu_0$ for all filament lengths, indicating that the filament could extend without a limit length.

c) The effect of varying the bending modulus C in the coarse-grained model. Larger C did not affect the predicted limit lengths, but decreased the predicted pitch angles.

d) The effect of varying the twist modulus K in the coarse-grained model. Larger K decreased the predicted limit lengths, but did not substantially alter the predicted pitch angles.

e) Increasing the membrane binding potential *V* increased the predicted limit lengths but did not alter the predicted pitch angles. The pitch angle values were highly overlapping for all values of *V*.

Supplementary References

1 Ouzounov, N. *et al.* MreB orientation correlates with cell diameter in Escherichia coli. *Biophysical journal* **111**, 1035-1043 (2016).

2 Colavin, A., Shi, H. & Huang, K. C. RodZ modulates geometric localization of the bacterial actin MreB to regulate cell shape. *Nature communications* **9**, 1280 (2018).

3 Shi, H. *et al.* Deep phenotypic mapping of bacterial cytoskeletal mutants reveals physiological robustness to cell size. *Current Biology* **27**, 3419-3429. e3414 (2017).
