# Supplementary material for: Chiral twisting in a bacterial cytoskeletal polymer affects filament size and orientation
Source: Nat Commun. 2020 Mar 16;11:1408. doi: 10.1038/s41467-020-14752-9 (PMC7075873; doi:10.1038/s41467-020-14752-9)
Supplement: Supplementary file 2 — Description of Additional Supplementary Information [file 41467_2020_14752_MOESM2_ESM.pdf]

Description of Additional Supplementary Files

File Name: Supplementary Movie 1: An 8x2 ATP-bound MreB protofilament bends and twists in water
Description: Equilibrium MD simulation of an 8x2 ATP-bound MreB protofilament in water. In this 60-ns simulation, the filament exhibits twisting and bending similar to the 4x2 protofilaments. The two single protofilaments are colored cyan and dark blue to visualize the effect of twist.

File Name: Supplementary Movie 2: A pre-twisted MreB protofilament untwists when placed onto a membrane patch.
Description: The equilibrated, twisted MreB double protofilament from a water simulation was placed ~10 Å away from a membrane patch. In the 120-ns simulation, the filament untwisted from the right end while “zippering” into the membrane. Cyan and dark blue ribbons are two single protofilaments composing the MreB double protofilament. Colored spheres are the membrane patch.
